# Response of Cellular Innate Immunity to Cnidarian Pore-Forming Toxins

**DOI:** 10.3390/molecules23102537

**Published:** 2018-10-04

**Authors:** Wei Yuen Yap, Jung Shan Hwang

**Affiliations:** 1 Department of Biological Sciences, School of Science and Technology, Sunway University, No. 5 Jalan Universiti, Bandar Sunway, Selangor Darul Ehsan 47500, Malaysia; wei.y58@imail.sunway.edu.my; 2 Department of Medical Sciences, School of Healthcare and Medical Sciences, Sunway University, No. 5 Jalan Universiti, Bandar Sunway, Selangor Darul Ehsan 47500, Malaysia

**Keywords:** actinoporin, cnidarian pore forming toxins, innate immune response, potassium efflux, inflammation

## Abstract

A group of stable, water-soluble and membrane-bound proteins constitute the pore forming toxins (PFTs) in cnidarians. They interact with membranes to physically alter the membrane structure and permeability, resulting in the formation of pores. These lesions on the plasma membrane causes an imbalance of cellular ionic gradients, resulting in swelling of the cell and eventually its rupture. Of all cnidarian PFTs, actinoporins are by far the best studied subgroup with established knowledge of their molecular structure and their mode of pore-forming action. However, the current view of necrotic action by actinoporins may not be the only mechanism that induces cell death since there is increasing evidence showing that pore-forming toxins can induce either necrosis or apoptosis in a cell-type, receptor and dose-dependent manner. In this review, we focus on the response of the cellular immune system to the cnidarian pore-forming toxins and the signaling pathways that might be involved in these cellular responses. Since PFTs represent potential candidates for targeted toxin therapy for the treatment of numerous cancers, we also address the challenge to overcoming the immunogenicity of these toxins when used as therapeutics.

## 1. Introduction

Cnidarians (jellyfish, sea anemones, sea pens, corals) are the most common venomous animals in the sea. Swimmers and divers can be stung when brushing against jellyfish or stepping on sea anemones or corals. Some, such as the Australian box jellyfish, *Chironex fleckeri*, contain a deadly venom that is fatal to humans. Others may not be fatal, but can cause mild to severe reactions to the venom, depending on the species, the amount of toxins and the immune system of the person. Clinical manifestation of envenomation can be local, systemic or fatal [1]. A local sting by venomous cnidarian results in burning pain and urticarial eruption at the site of contact with the animal. The pain is caused by the action of neurotoxins on cutaneous sensory nerves, while the rash is due to the innate immune response mounted against the exogenous toxins. A local response can become severe and last for weeks if not treated properly. Russo et al. reported a case in which an individual was found to develop a large, local edematous response after encountering the jellyfish, *Chrysaora quinquecirrha* and *Pelagia noctiluca*. The response lasted for 10 to 14 days with a high antigen-specific IgE level to sea nettle and man-of-war antigen in the body [2]. Unlike local cutaneous eruption, a systemic reaction causes symptoms such as headaches, chest pain, muscle pain, sweating and nasal water discharge. These symptoms indicate a constitutional toxic reaction to the venom. The strongest reaction to cnidarian venom is rather fatal and causes anaphylaxis, an allergic reaction that may lead to death. The word “anaphylaxis” was created by Charles Robert Richet who was the professor of physiology in the Faculty of Medicine at the Collège de France in Paris. Charles Richet discovered a phenomenon of life-threatening allergic reaction when he re-injected extracted toxins from *Actinia equina* into a dog. The first dose of extracted toxins was not strong enough to kill the dog. However, when the dog was injected with lower dose of the same toxins 3–4 weeks after the first injection, the dog developed vomiting, blood diarrhoea, syncope, unconsciousness, asphyxia and finally died. This phenomenon was later termed “anaphylaxis” and Richet was awarded the Nobel Prize in 1913 for his research on this topic. Our current knowledge suggests that, during anaphylaxis, there is an acute reaction of mast cells and basophils which are the key players in allergy [3]. Degranulation of these two immune cells releases granule-associated, pro-inflammatory molecules such as histamine, tryptase, chymase, heparin and cytokines that are involved in the degradation of tissue. This leads to “shock organ” characterized by low blood perfusion to cellular tissues and damage of organs [3].

As can be seen from the above, cnidarians have been studied extensively in the context of envenomation and toxicology, revealing insights into many important biological phenomena [4,5,6]. Despite this biological perspective, several of the most intriguing aspects of these organisms remain unanswered. How are such morphologically simple, soft organisms able to produce lethal toxins that kill a dog? Do these toxins share an evolutionally conserved function with toxins of other organisms? How does the human immune system react to cnidarian toxins? It is evident that answers to these questions can be obtained from the studies of human or mammalian innate immune reactions to toxins. These questions bring us to the main rationale for the current review, which discusses various cnidarian pore-forming toxins and the cellular immune responses to these toxins. Furthermore, the review addresses the innate immune response to the toxins with an emphasis on the development of toxin therapeutics.

## 2. Diverse Toxins Targeting Same Membrane Receptors

Toxins are commonly found in both prokaryotes and eukaryotes. They are used for prey capture and defense and to respond to environmental competition [7]. The current forms of toxins have evolved for millions of years through adaptation in order to favor three basic functions: physical immobilization, blood anticoagulation and inflammation, which span the range of severity from mild to lethal [7]. However, the study of toxin is not only about understanding the evolution and adaptation of organisms towards the environment but also understanding human pathophysiology and defense mechanisms. Toxins can directly target human tissues. Many pathogenic bacteria such as *Salmonella typhi*, *Bacillus cereus*, *Neisseria gonorrhoeae*, *Staphylococcus aureus* and *Mycoplasma pneumoniae* select their human host due to nutrient-rich, damp and warm environment [8]. Other poisonous organisms such as insects, snakes, frogs and some marine organisms also secrete toxins that target a diverse range of predators including human. Therefore, same toxins must be able to act against a variety of organisms and interact with common target sites shared between organisms. This is one way to allow toxins to be produced economically since toxins are protein-rich substances which require extensive, complex metabolic and biochemical pathways [9]. These common target sites are located on the cell membrane, they can be lipids, glycolipids, glycoproteins, integral or peripheral membrane proteins. Depending on the kind of toxin, functionally distinct groups of toxins recognise target sites that are specifically present on certain type of cells such as neurons. All neurotoxins secreted by poisonous organisms have a destructive action against the nervous system. Nicotinic acetylcholine receptor (nAChR), for instance, is the target of wide variety of neurotoxins [10]. nAChR is a ligand-gated ion channel present predominantly in the plasma membrane at the neuromuscular junction. It has a binding site for the neurotransmitter acetylcholine. Upon binding of acetylcholine to the nAChR, a conformational change occurs in the receptor and causes a rapid opening of a channel that allows the passage of Na^+^, K^+^ and Ca^2+^ into the cell. The flow of cations subsequently induces membrane depolarization and contraction of muscle [11]. One of the α-neurotoxins, α-Bungarotoxin, which is isolated from the krait snake *Bungarus multicinctus*, binds to the α-subunit of muscular nAChR and inhibit acetylcholine from binding to nAChR. Other similar α-neurotoxins of Elapid snakes, α-conotoxins of cone snails and lophotoxins of corals [12,13] also block the binding of acetylcholine to nAChR, suggesting that these neurotoxins have independently evolved into the antagonist of nAChR and used the same mechanism to disrupt the acetylcholine transmission. 

## 3. Pore-Forming Toxins

The best studied models of pore-forming toxins (PFTs) come from bacteria since the bacterial PFTs are the largest class of bacterial virulence factors and also the best characterized PFTs. We shall here describe the basic properties and examples of bacterial PFTs and more about cnidarian PFTs in other sections below. Table 1 also provides the list of PFTs described in this review. Several classes of pathogenic microorganisms cause infectious diseases by producing pore-forming toxins to attack host cells. To make pores in the plasma membrane, pore-forming bacteria produce two types of PFTs, α- and β-PFTs [14]. 

This classification is based on the secondary structure of the membrane spanning region, which induces pore formation. α-PFTs such as the PFTs of the colicin family, ClyA family and actinoporin family form pores using clusters of α-helical monomers while β-PFTs such as the PFTs of hemolysin family, aerolysin family and cholesterol-dependent cytolysins (CDCs) family form pores by formation of transmembrane β-barrels [48,49]. Prior to the formation of the pore, these PFTs establish contacts with the plasma membrane via the protein-lipid, protein-glycan or protein-protein interactions [50,51]. Many bacterial and sea anemone PFTs use the abundant and common membrane constituents, sphingomyelin or cholesterol, as binding sites. Actinoporin, a small α-PFT (18–22 kD), produced by a number of sea anemones, uses a β-sandwich fold with exposed aromatic amino acids for specific attachment to the plasma membrane [52]. The β-sandwich fold consists of a cluster of conserved tryptophan or tyrosine residues with protruding the aromatic rings that forms a combination of hydrophobic and electrostatic interactions with the membrane [53,54]. 

Hemolysins are β-PFTs and they play a major role in bacteria pathogenesis. For instance, three distinct hemolysins of *Staphylococcus aureus*, α-hemolysin (Hla), γ-hemolysin (Hlg) and leukocidins (e.g., HlgACB, LukED), kill various cells of the immune system and promote bacterial dissemination to cause pneumonia and sepsis [55]. They bind specifically to their respective receptors on the surface of immune cells [17,56]. α-hemolysin (Hla) binds to phosphocholine and disintegrin and to the metalloproteinase domain-containing protein 10 (ADAM10) on epithelial cells [57,58]. Binding of Hla to three different receptors causes the formation of heptameric pores on the epithelial cells, thus disrupting the epithelial barriers through E-cadherin proteolysis [59]. The γ-bi-component hemolysins AB and CB (HlgAB, HlgCB) bind to the chemokine receptors CXCR1, CXCR2 and CCR2 to target neutrophil and to the complement receptors C5aR and C5L2 to target monocytes [60]. LukED targets macrophages, dendritic cells and T cells by binding to CC-chemokine receptor type 5 (CCR5) or neutrophils and monocytes by binding to CXC-chemokine receptor type 1 (CXCR1) and CXCR2 [61,62,63].

## 4. Pore Forming Mechanism in Host Cell Membrane

The molecular mechanism of pore formation can be summarized into four major steps: (1) membrane binding, (2) oligomerization, (3) membrane insertion and (4) cell death. (1) It begins with the production and the secretion of PFTs in a water-soluble monomeric protein form, which then undergo confirmation changes to the membrane-bound state upon binding to sugars, lipids or receptor proteins in the target cell membrane. In rare situation, PFTs may bind non-specifically to the cell membrane [64,65]. (2) Following membrane binding, the monomeric proteins assemble into an oligomeric form with a ring-like structure. (3) For most β-PFTs, the oligomerization takes place at the surface of the cell membrane before the insertion of β-strands into the cell membrane. By contrast, the majority of α-PFTs undergo a conformation change with part of the polypeptide chain inserting into the lipid bilayer and simultaneously oligomerizing. (4) As the number of pores or channels increases, the cell undergoes cell death [65,66,67,68,69,70]. Bacteria PFTs are especially well studied for their molecular mechanism of pore formation that leads to cell death. Colicin of *E. coli* is a good example. It is used to kill competing bacteria. Colicin forms pores on the target cell by insertion of a hydrophobic helical hairpin into the inner plasma membrane that leads to membrane depolarization, ATP depletion and cell death [18,71,72].

Different PFTs cause different degrees of membrane permeability, which will lead to different response of the target cells [73]. However, the extent of membrane permeability also varies depending on the pore produced by the PFT. Some PFTs allow only specific ions, such as potassium and/or calcium, to pass through. While other PFTs allow small molecules such as ATP, or larger molecules such as proteins to pass through [66]. Therefore, characterization of the pore produced by a specific PFT is required to fully understand the function of a PFT. 

## 5. Cnidarian PFTs

The complete spectrum of cnidarian toxins is not well known but it comprises a variety of compounds such as pore-forming toxins (neurotoxins and cytolysin toxins), enzymes (phospholipase A2 and metalloproteases), and vasodilatory biogenic amines (serotonin, histamine, bunodosine and caissarone) [4,74]. However, we will only discuss the cnidarian PFTs in this article. Cnidarian PFTs are broadly cytolytic and have been identified in all Classes in the phylum Cnidaria including jellyfish, sea anemones and hydroids [75]. To date, the function of cnidarian PFTs has not been studied in detail and hence the best way to distinguish them is based on their molecular weights as described in Anderluh et al. (2011) [76]. In this article, we divide the cnidarian PFTs into actinoporins (20–22 kD), hydralysin-related toxins (27–31 kD), jellyfish toxins (42–46 kD) and membrane-attack complex/perforin family (MACPF) (60 kD).

### 5.1. Actinoporins (20–22 kD)

The first report of actinoporins came from a study of a cytolytic toxin isolated from *Actinia equina* [77]. Actinoporins are α-PFTs produced by many sea anemone species such as *Stichodactyla heliantus* (sticholysin), *Actinia equina* (equinatoxin) and *Actinia fragacea* (fragaceatoxin C) [31,33,77,78,79]. They are 20–22 kDa highly basic proteins with an isoelectric point (PI) above 9, which lack cysteine residues, and are dependent on sphingomyelin for membrane binding [65,80]. Based on structural analysis, actinoporins have a tightly folded core of β-sheet strands with two short α-helices on each side of β-sheets [65,80,81]. They have a conserved tryptophan domain and an Arg-Gly-Asp (RGD) motif that are required for the initial recognition and binding to receptors (sphingomyelin or cholesterol) on the cell membrane and an N-terminal α-helix for pore formation [65,81,82,83]. The oligomerization of 3 or 4 monomers can induce a pore size of 1–2 nm in diameter [82,84].

The best known actinoporins are equinatoxin II (EqtII) of *Actinia equina* and sticholysin II (StII) of *Stichodactyla helianthus* [85,86]. Recently, Bellomio et al. (2009) have discovered fragaceatoxin C (FraC) of *Actinia fragacea* [33]. Cnidarians use actinoporins to paralyse prey and defend against predators. Actinoporins bind specifically to sphingomyelin in the cell membrane and cause cell lysis by forming pores in the membrane of target cells. Actinoporin-like toxins were also found in *Hydra magnipapillata* [34]. Seven actinoporins have now been identified and they are named as *Hydra* actinoporin-like toxin (HALT) 1–7. Among the seven HALTs, HALT-1 is the only toxin that has been shown to be hemolytic and cytolytic against human cells [34,87]. 

### 5.2. Hydralysin-Related Toxins (27–31 kD)

Hydralysin (Hln) was first isolated from *Chlorohydra viridissima*, a green *Hydra* which contains symbiotic *Chlorella* in endodermal cells. It is a β-PFT sharing sequence homology with aerolysin from *Aeromonas* spp. and α-toxin from *Clostridium* spp., cytolysin LSL (from fungus *Laetiporus sulphureus*), parasporin-2 (from bacterium *Bacillus thuringiensis*) [22,88]. Its cytotoxic activity is cell-type selective, effectively lysing insect cells but not mammalian cells.

Hydralysin was shown to be paralytic and neurotoxic for ingested preys in the gastrovascular cavity of *Hydra* [35,89]. Since Hln does not recognize mammalian membrane lipids or glycans, it must target either an analogous lipid or glycan or a specific membrane protein. This is also supported by the fact that Hln also does not bind to *Hydra* cell membranes, suggested that the role of Hln in *Hydra* digestion system is determined by it specific binding site on its target. A hydralysin-related toxin, Nvlysin-1b, was found in the sea anemone, *Nematostella vectensis,* and shown to be expressed in large ectodermal cells (likely gland cells) in the pharynx [36].

### 5.3. Jellyfish Toxins (42–46 kD)

These toxins are very potent cytolysins, isolated from the box jellyfish species *Carybdea rastoni*, *Carybdea alata*, *Chiropsalmus quadrigatus*, and *Chironex fleckeri* [37,38,39,45]. Most of these cubozoan pore-forming toxins show potent cytotoxic activity and lethality to mice, crayfish, and human, as well as, hemolytic activity to sheep erythrocytes. Nagai et al. (2000) identified two toxins in *C. rastoni* toxin-A and toxin-B (CrTX-A and CrTX-B) of 43 and 46 kDa, respectively [37]. Immunolocalisation studies on *C. rastoni* toxins indicated that CrTX-A is mostly localized within the nematocysts (stinging organelles specific to Cnidaria), whereas CrTX-B is found in tentacle tissue. However, only a single cDNA encoding both proteins (CrTXs) was cloned and sequenced, suggesting that CrTX-B is synthesised in tentacle tissue, modified to CrTX-A, and subsequently incorporated in nematocysts [37]. Based on the secondary structure prediction, this group of jellyfish toxins are likely α-PFTs [90].

Studies on *C. alata* similarly showed that, following the purification of CaTX-A and -B from *C. alata* tentacles, only CaTX-A (not CaTX-B) was localized to the nematocysts [38]. Two years later, the major protein contained in *C. quadrigatus* nematocyst venom (CqTX-A) was isolated, cloned and sequenced [39]. Although *C. quadrigatus* was reputed to be more dangerous to humans than *C. rastoni* and *C. alata*, comparisons of the lethal and hemolytic activity between all isolated toxins from these three species revealed that CqTX-A was less potent than CrTX-A and CaTX-A [39]. However, this apparent contradiction can be explained by the fact that *C. quadrigatus* has more and longer tentacles than *C. rastoni* and *C. alata*, giving *C. quadrigatus* the potential to inject larger doses of toxins than the smaller, four-tentacled carybdeids [45].

In contrast to the other species of box jellyfish, two homologous proteins (CfTX-1 and -2) were isolated from the nematocysts of *C. fleckeri* [45]. This study clearly indicated that unlike CrTX-A and -B, the two proteins are not encoded by the same gene since two distinct cDNA were isolated. In addition, the hemolytic activity of the CfTX proteins exhibited a sigmoidal dose-response curve, suggestive of a stoichiometric cytolysin characteristic [45].

In another jellyfish, *Malo kingi*, known to cause Irukandji syndrome, two cytolysins, MkTX-A and MkTX-B, homologous to those previously reported were identified [40]. Further characterisation revealed high expression levels of these genes but localisation studies by *in situ* hybridisation techniques indicated their putative involvement in the box jellyfish defense system. 

Several bioinformatic analyses reported high sequence homology between the five cubozoan toxins (CrTX-A, CaTX-A, CqTX-A, CfTX-1 and -2). Based on secondary structure predictions, at least two common structural features have been identified and may relate to the potent cytolytic activity of the proteins. Firstly, an amphiphilic α-helix is predicted in the N-terminal region of each protein [45] and secondly, a common transmembrane-spanning region (TSR1) is predicted in the cubozoan sequences, which coincides with several highly conserved amino acids [45]. Furthermore, remote protein homology analyses predicted that two of the cubozoan proteins (CaTX-A and CfTX-2) share weak structural similarity to α-PFTs produced by *Bacillus thuringiensis* [45].

### 5.4. MACPF (60 kD)

Sea anemones were found to possess proteins similar to the membrane-attack complex/perforin (MACPF) family. MACPF was first studied in the complement system of the host innate immune system. It was discovered later that a number of cnidarian PFTs also contain the domain of MACPF. MACP-containing toxin was discovered in nematocysts of the stinging sea anemone *Phyllodiscus semoni* [91]. The 60 kDa MACPF cytolysins (PsTX-60A and PsTX-60B) were lethal to shrimps (LD_50_ < 800–900 μg/kg) and hemolytic (EC_50_ < 600 and < 300 ng/mL) against sheep erythrocytes [91]. Although similar to perforins, these cytolysins lack the conserved C2 domain responsible for attachment to lipid membranes [76]. AvTX-60A of the sea anemone *Actineria villosa* was also identified as the orthologue of PsTX-60A/B [43]. In addition, two *Hydra* proteins, HyMac and apextrin, also contain MACPF domain [44]. It has a typical β-pore forming structure when inserted into the cell membrane.

## 6. Activation of Innate Immunity by Pore-Forming Toxins

Pathogenic bacteria produce PFTs to damage host tissue, either by promoting colonization or by protecting itself from the host defense system. Being foreign or non-self molecules, PFTs trigger the innate immune system of the host. The role of bacterial PFTs in the host immune response has been studied extensively in the last decades. Basically, the damaged cell can either be repaired by restoring the integrity of plasma membrane or undergo cell death via necrosis or apoptosis [66,73]. Which host cell responses takes place upon PFT induction is primarily determined by the host cell-type, the type of PFT, the toxin concentration and the properties of pores [92,93,94]. The following sections describe the human innate immune responses to cnidarian PFTs and the signaling pathways that are commonly activated by bacteria PFTs.

Similar to bacterial PFTs, cnidarian PFTs can induce the innate immune response via the cellular potassium (K^+^) efflux. The cytosolic concentration of potassium is approximately 139 mM, which is much higher than other ions such as sodium (12 mM), chloride (4 mM) and calcium (<0.0002 mM). When PFTs puncture the plasma membrane, they cause K^+^ efflux and reduce intracellular K^+^ concentration. Depletion of intracellular K^+^ and also changes in other ion concentrations activate the inflammatory signaling pathway. This has been described by Nagai whereby the intraperitoneal injection into mice of 100 µg/kg of CrTX-A from the box jellyfish, *Carybdea rastoni*, will kill them in 8 h; and smaller amounts of CrTX-A (0.1 µg/injection) resulted in cutaneous inflammation which is similar to a *C. rastoni* sting on human skin [37]. The results suggested that the formation of pores by CrTX-A induces a cascade of signaling reactions leading to the acute inflammatory lesion [37]. A similar study by the same group of researchers identified a hemolytic extract containing CaTX-A from *C. alata* and showed that this CaTX-A could act as an inflammatory factor, although it is less potent than CrTX-A of *C. rastoni* [38]. Both CrTX-A and CaTX-A are orthologous in amino acid sequence and have the same estimated molecular weight of 43 kDa. Other amino acid sequence othologs have the same estimated molecular weight of 43 kDa. Two other othologous toxins from *Chironex fleckeri*, CfTX-1 (~51.4 kDa) and CfTX-2 (~51.7 kDa), were co-purified using size exclusion chromatography and cation exchange chromatography [46,95]. Both CfTX-1 and -2 have high hemolytic activity to sheep erythrocytes [95] but their potencies were much lower than the other two PFTs, CfTX-A (~40 kDa) and CfTX-B (~42 kDa), which were also isolated from *Chironex fleckeri* [46]. Until now, there is no direct evidence of molecular association between cellular responses and jellyfish toxins, or whether inflammation occurs in toxin-treated immune cells or animals. Yet, it is quite apparent that acute inflammation often occurs after a jellyfish sting and repeated stings may lead to anaphylaxis. A case study of a 59 year-old woman, who was stung by an unknown jellyfish in Greece, documented this response. When she was stung a second time, she immediately suffered from shortness of breath, oedema, unconsciousness and extreme hypotension [96]. These are the symptoms of anaphylaxis. High levels of histamine and IgE were detected in basophils that were isolated from the patient [96]. Histamine is largely produced by mast cells and basophils during inflammation, and is a hallmark of both innate and adaptive immune responses. Moreover, the elevated IgE level indicated that the jellyfish toxins were the allergens which induced the adaptive immune system via the mast cells and basophils.

Membrane Attack Complex of Complement and Perforin (MACPF)—containing toxins have been identified in *Phyllodiscus semoni* (PsTX-60A and PsTX60B) and *Actinaria villosa* (AcTX-60A) [41,42,43]. Thus far, no information is given on the molecular mechanism of these sea anemone toxins on the attack of host cells, but we collected relevant information of the functional characteristics of these MACPF-containing toxins. AvTX-60A is evolutionarily orthologous to PsTX-60A and PsTX-60B [43]. They all are originated from the nematocysts, having the MACPF domain at the N-terminus and exerting cytolytic and hemolytic effects on their target cells. No direct evidence indicates that MACPF-containing toxins cause the innate immune responses. However, symptoms such as inflammation and tissue necrosis are often observed after sea anemone stings. A case reported that a Japanese adult developed dermatitis with severe swelling and ulceration at the site of contact and acute kidney injuries including cytolysis and hemolysis in multiple renal tissues after he accidentally touched *P. semoni* [97]. Venom extracted from *P. semoni* has also shown the nephrotoxicity in rat after the administration of 0.03 mg of venom [98].

## 7. Molecular Mechanism of Cellular Immune Responses to Actinoporin

Actinoporins are well-defined α-pore-forming toxins that are only found in the phylum of Cnidaria. They are found abundantly in sea anemones, where more than a dozen actinoporins or actinoporin-like proteins have been identified. More than half of them have multiple isoforms [99]. Two actinoporins, sticholysin II and equinatoxin II, have been well investigated structurally by X-ray crystallography and NMR spectroscopy and functionally characterized by mutational and cytolytic analyses. However, many questions still remain to be answered, e.g., whether cell lysis or necrosis is the only mechanism to cause the cell death? Can cells recover from the damage to the cell membrane? Do cells react differently to various concentrations of actinoporin? To answer these questions, we can get some clues from molecular studies of the cellular responses to bacterial PFTs. Due to the formation of pores, bacterial PFTs induce the potassium (K^+^) efflux followed by downstream signaling cascades. It has been shown that two signaling cascades, the NLRP3 inflammasome and p38 MAPK, are induced following a drop in cellular K^+^ concentration. Autophagy, an intracellular process for the degradation of cytoplasmic contents, can be triggered by bacterial PFTs but likely not engage any signaling cascades. Autophagy keeps the cytoplasmic contents in a doubled-membrane vesicle-autophagosome and then fusing the autophagosome with lysosomes for degrading the contents. This mechanism has been used to protect the host cell against pathogenic bacteria or particles [95,100,101] or indirectly promote the host cell death [102].

### 7.1. Activation of MAPK Pathways by Actinoporins

**Figure 1:**
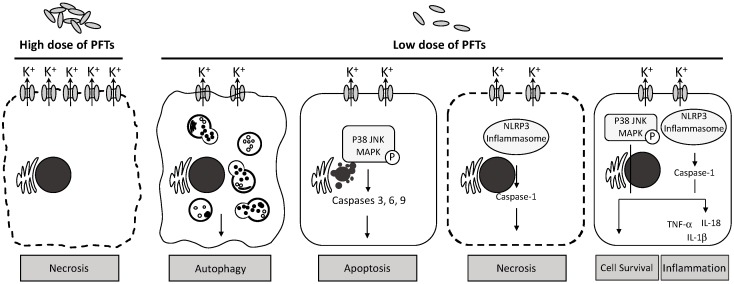
Host cellular responses to the low and high concentrations of cnidarian PFTs. Pore formation of cnidarian PFTs may lead to difference cellular responses in host cells. At high doses of PFTs, multiple pores in the cell membrane cause an imbalance between intra- and extracellular osmotic pressures, resulting in sudden cell burst. Lower doses of PFTs can induce the intrinsic defense mechanism—autophagy that promotes intracellular engulfment and degradation. If the damage to the cell membrane cannot be repaired, the cell may undergo either programmed cell death (apoptosis) or NLRP3-dependent programmed necrosis, through p38 MAPK or NLRP3 inflammasome pathway, respectively. These two signaling pathways are also involved in either the cell survival with the restoration of intracellular osmotic environment or the activation of the cellular innate immune response, releasing pro-inflammatory proteins. In certain circumstances, inflammation is followed immediately by apoptosis or necrosis.

Cabezas et al. investigated the intracellular K^+^ concentration when StII was applied to Baby Hamster Kidney (BHK) cells and the recovery of K^+^ homeostasis afterward [92]. The authors found that depletion of intracellular K^+^ occurred immediately after BHK cells were treated with StII. However, the survival of BHK cells and the restoration of K^+^ homeostasis was StII concentration-dependent. When a high concentration of StII such as 15 nM was used, damage to the cell membrane was too large and led to cell death (Figure 1). At lower dose of StII, the authors showed that the K^+^ efflux was able to induce the MAPK pathways, as shown by the activation of p38 and Erk1/2. This is the first case showing that actinoporins act similarly to bacterial PFTs and induce pMAPK pathways, one of the classic signaling pathways in the innate immune system [92] (Figure 1). A separate report also suggested that StII is capable of stimulating the pMAPK pathways and hence causing a totally different cellular response than necrosis [103]. In this case, the authors detected the release of Ca^2+^ from ER in Raji cells. The changes of the cytosolic Ca^2+^ level were directly linked to the pMAPK pathways as well as to the expression of Receptor Interacting Protein 1 (RIP1). Interestingly, apoptosis was not observed, suggesting that StII pore formation did not lead to apoptosis [103]. On the other hand, RTX-A, an actinoporin of sea anemone *Heteractis crispa*, causes apoptosis at low concentration (<0.25 nM) while inducing necrotic effects at high concentration (<1 nM) [47]. These results suggested that the cellular response to cytotoxic activity of actinoporins is not only dose-dependent but also dependent on the type of actinoporin.

The mitogen-activated protein kinase (MAPK) cascades serves as a first-line defense mechanism in host cells. The human MAPK cascades plays a pivotal role in activating defense mechanisms against bacterial PFTs [104]. Some, such as extracellular signal-regulated kinase 1 (ERK1), ERK2, p38α, Jun N-terminal kinase 1 (JNK1) and JNK2 have been extensively studied in the context of innate immunity. The p38 MAPK signaling cascade was first demonstrated in the nematode *C. elegans* to have a role in regulating the innate immune response to the PFT Cry5B of *Bacillus thuringiensis* [105]. Nematode mutants lacking p38 MAPK became more susceptible to killing by bacterial pathogens [105]. At least four genes from *C. elegans* are involved in the p38 MAPK cascade and their human orthologues have been identified. The genes *nsy-1, sek-1* and *pmk-1* encode the *C. elegans* orthologues of human ASK-1 MAPK kinase kinase (MAPKKK), MKK3/MKK6 MAPKK, and p38 MAPK, respectively, all of which have been directly implicated in the mammalian cellular immune response [106]. PMK-1 is likely to be phosphorylated by NSY-1, and NSY-1 is phosphorylated by SEK-1. Another *C. elegans* gene *tir-1* encode a scaffold protein which functions upstream of NSY-1, SEK-1 and PMK-1, and has its homologous counterpart, sterile α- and armadillo-motif-containing protein (SARM), in human [107].

### 7.2. Does Actinoporin Activate NLRP3 Inflammasome?

To date, many pathogenic bacteria have been shown to activate the host innate immune response by secreting toxins and influencing the concentration of K^+^ in the cytosolic compartment. These toxins include α-hemolysin (or α-toxin) of *Staphylococcus aureus*, aerolysin of *Aeromonas hydrophila*, tetanolysin O of *Clostridium tetani*, pneumolysin of *Streptococcus pneumonia*, and etc. [27]. Muñoz-Planillo et al. demonstrated that depletion of cytosolic K^+^ is the main key factor responsible for the activation of NLRP3 compared to other stimuli such as mitochondrial perturbation, ROS production, change in cell volume, and membrane permeability to organic molecules and ions [108]. NLRP3 (NACHT, LRR and PYD containing protein) consists of three domains: NACHT as the central nucleotide-binding and oligomerization domain, LRR as C-terminal leucine-rich domain and PYD as N-terminal caspase recruitment (CARD) or pyrin (PYD) domain [109]. In the presence of ATP, NLRP3 then recruits apoptosis-associated speck-like protein containing a CARD (ASC) and caspase-1. Activated caspase-1 is able to cleave pro-interleukin-1β and pro-interleukin-18 to their active form interleukin-1β (IL-1β) and interleukin-18 (IL-18). Both IL-1β and IL-18 are cytokines and they are released from immune cells (e.g., macrophages) to further enhance the proinflammatory immune response [110]. Although we know the important role of the NLRP3 inflammasome in the innate immune response induced by the depletion of intracellular concentration of K^+^, the exact mechanism of how the level of K^+^ regulates the expression of NLRP3 per se and the formation of NLRP3 inflammasome is still unclear (Figure 1). A clue to the formation of NLRP3 inflammasome was obtained in a recent study by Stutz et al. which showed that assembly of inflammasome depended on the phosphorylation in the PTD domain [111].

Although the NLRP3 inflammasome is known to be involved in the innate immune response following cell membrane disruption by bacterial PFTs, the role of actinoporins in the formation of the NLRP3 inflammasome has not yet been reported. As mentioned above, sticholysin II (StII) creates membrane pores and alters cytosolic ion composition that lead to the induction of the R1P1-MEK1/2, ERK1/2 and MAPK pathways. These pathways act in turn to restore cellular homeostasis and subsequently the closure of pores. However, we do not know if the changes in cytosolic ion composition caused by StII can also induce formation of the NLRP3 inflammasome. This is yet to be investigated.

### 7.3. Signaling Pathways via Pattern Recognition Receptors

Besides the p38 MAPK and the NLRP3 inflammasome, the third signaling pathway involves pattern recognition receptors (PRR) which are commonly found on the plasma membrane, endosomal membrane or cytoplasm [112]. Pattern recognition receptors (PRR) include Toll-like receptors (TLRs), C-type lectin receptors (CLR), Nod-like receptors (NLRs), and retinoic acid inducible gene (RIG)-I-like receptors. Each PRR binds to its cytoplasmic ligands or proteins of the respective signaling cascade. Toll-like receptor 4 (TLR4), for instance, is the first human immune receptor to be identified and is recognized by LPS (lipopolysaccharide) of Gram-negative bacteria, hence activating the host innate immune system. TLR4, when bound to LPS, is present as a dimer on the surface of the plasma membrane of macrophages, and a conformational change of TLR4 lead to the dimerization of TIR (Toll IL-1 Receptor) in the cytoplasm. TIRs are associated with the adaptors MyD88 [113] and TRIF and activate a series of downstream cascades for the expression of NFkB (Nuclear Factor Kappa B) and thus to pro-inflammation (release of cytokines and Chemokines). So far, no evidence shows a direct recognition of PRR by actinoporins, however, we cannot rule out the possibility that actinoporins trigger the IL-1β/IL-18-MyD88 pathway via PRR as a secondary or redundant mechanism. In fact, *Vibrio cholera* cytolysin (VCC), a bacterial β-PFT binds TLR2/TLR6 as a membrane receptor and recruits MyD88 in the presence of intracellular TIRAP. After that, MyD88 initiates downstream signaling cascades via the formation of IRAK1/IRAK4/TRAP6 complex, activating the expressions of proinflammatory cytokines including TNF-α and IL-6 [114]. TLR2/TLR6 is not the only pathway manipulated by VCC. VCC can form anion-selective pores by binding to cholesterol in the plasma membrane, thus either causing necrotic cell death or inducing membrane damage [115]. VCC also induces apoptosis via a TLR-independent pathway [116], suggesting that there are multiple cellular responses to VCC.

## 8. What Have We Learnt So Far and How Do We Move Forward?

The responses of the immune system towards cnidarian actinoporins involve complex mechanisms that include a broad range of cell types, intertwined protein-protein networks and multiple signaling pathways. As we have discussed above, only a few reports are available on the immunogenicity of actinoporins. All early studies of cnidarian toxins were based on clinical diagnosis of patients, who were stung by either jellyfish or sea anemones while they were swimming in a jellyfish or sea anemone infested water [117,118]. Burning, pain, prickling, rash and edema are typical signs of inflammation and hence it was concluded that the venom of jellyfish or sea anemone is able to induce the innate immunity. The first evidence that directly demonstrates the involvement of humoral and cellular immunity towards actinoporin was described by Narat et al. [119]. Following venom injection, the authors were able to identify specific antibodies to Eqt using an ELISA assay. However, the antibodies were not able to completely protect erythrocytes from hemolysis [119]. Not until recently, Cabezas et al. have shown that StII can generate a cellular response involving K^+^ efflux and activate the p38 MAPK (mitogen-activated protein kinase) pathway [92]. Unlike the classical view that actinoporins only physically lyse cells, the role of actinoporins can now be diversified as based on the dosage of toxin, the host cell type and the availability of receptor on the cell membrane. This suggests that all actinoporins may trigger the innate immune response in a similar manner to that mediated by bacterial PFTs. As such, actinoporins could trigger a sequence of reactions that may result in necrosis, apoptosis or cell survival. As we have known thus far, all actinoporins can lyse erythrocytes [87,120,121]. In these experiments, the underlying mechanism that leads to hemolysis appears to be necrosis, since erythrocytes lack nuclei and mitochondria and have been thought to possess limited capacity to undergo apoptosis [122]. These experiments require high doses of actinoporins. The mechanism of cell death at lower doses of toxin is potentially different. Some PFTs, if they do not completely rupture the cell membrane, may internalize into the cell and induce apoptotic cascades. These PFTs include α-toxin (or α–hemolysin) of *Staphylococcus aureus*, aerolysin of *Aeromonas hydrophila*, HlyA of *E. coli*, ALO of *Bacillus anthracis*, VCC of *Vibrio cholera*, RTX-A of *Heteractis crispa* and many more [47,49,116,123,124,125]. Taking *Staphylococcus aureus* α-toxin as an example, it was initially named α–hemolysin because of its rapid damage to erythrocytes [126]. It has also been found to exert necrotic action in other cell types [127,128], as well as NLRP3-dependent pyronecrosis in macrophages [129]. The mechanism of pyronecrosis is dependent on the activation of inflammation via the NLRP3 inflammasome (Figure 1). Interleukin-1β (IL-1β) was released when human monocyte-derived cells were exposed to α-toxin, and the induction of IL-1β was mediated by the NLRP3 inflammasome, although it is still unknown how this inflammasome-mediated signaling can lead to programmed necrosis [129,130]. Currently, to the best of our knowledge, pore formation by cnidarian actinoporins reduces the intracellular K^+^ concentration, which in return triggers the different cellular responses as mentioned above. Each cellular response could be mediated by pro-inflammatory cytokine signaling pathways such as NLRP3 inflammasome and p38 MAPK signaling cascades.

## 9. Cnidarian Pore-Forming Toxins for Targeted Toxin Therapy

Since PFTs kill cells effectively, they become potential candidates for targeted toxin therapy for the treatment of cancers and immune diseases [131,132]. Targeted toxin therapy, or it is now commonly named as immunotoxin, requires a targeting moiety to bring the toxin to target cells and to bind specific receptor or epitope on the target cells. The targeting moiety of immunotoxin is generally monoclonal antibodies, however, other proteins such as cytokines and growth factors have also been used as the targeting moiety [133,134,135,136,137,138]. In recent years, single chain fragment variable (scFv), which is created by linking the gene of variable fragment of the light chain (V_L_) and the gene of variable fragment of the heavy chain (V_H_) of an antibody with a flexible peptide linker consisting of glycine and serine residues, has gradually replaced monoclonal antibodies as the targeting moiety of immunotoxin. 

Currently, the therapeutic efficacy of immunotoxin is facing three major challenges—penetration ability, binding specificity and immunogenicity. Most immunotoxins constructed to date are intended for anticancer therapy. However, these immunotoxins are more effective towards haematological malignancies compared to solid tumors due to their poor tissue penetration ability. The main cause of limited penetration capability of immunotoxins is the molecular size of the immunotoxin. Toxins such as *Pseudomonas* exotoxin A mutant (38 kDa) and mature diphtheria toxin (58.3 kDa) commonly used as targeting moiety in immunotoxin are large in size with molecular weights ranging from 30–58 kDa [47,104,105]. Although they are effective in killing the target cells, the large size of these toxins leads to poor tissue penetration. In order to tackle this problem, smaller fractions of antibody such as scFv were considered. A full sized monoclonal antibody has a molecular weight of approximately 150 kDa. On the other hand, scFv, the smallest engineered fragment of an antibody, has a molecular weight of 25–30 kDa. The use of scFv in immunotoxin showed the improvement of tissue penetration in solid tumors. However, scFv has a lower binding affinity comparing to full sized mAb due to its monovalency. To solve this problem, tandem bivalent scFv or diabody scFv (50–60 kDa), triabody and fusion minibodies (80–105 kDa) were engineered. The binding affinity of bivalent scFv is comparable to the binding affinity of whole mAb although it has only less than half the molecular weight of a whole mAb [139].

The size of the immunotoxin can be further reduced by choosing the toxin with a relatively smaller molecular weight. This can be done by exploring other types of toxins. Studies have shown that the actinoporins derived from sea anemone could be used in the development of classical immunotoxin [140,141]. The size of these actinoporins is smaller (18.5–20 kDa) than other toxins used in conventional immunotoxin, and thus they should have a better tissue penetration capability. Avila and colleagues (1988) pioneered the first attempt of using actinoporins as the toxin moiety of an immunotoxin [142]. They chemically conjugated the hemolytic toxin (18 kD) of sea anemone *Stichodactyla helianthus* to mAb which is specific to IOR-T6 (an antigen expressed on immature T-lymphocyte). Similarly, when Avila et al. (1989) conjugated the same hemolytic toxin to a different monoclonal antibody directed against carcinoembriogenic antigen (CEA), the immunotoxin was highly toxic to CEA-expressing human breast carcinoma cells and not toxic to CEA-non-expressing human breast carcinoma cells [143]. In 1995, Pederzolli and colleagues conjugated equinatoxin II (EqtII) to transferrin to target transferrin receptor expressed on variety of tumor cells [144]. The study demonstrated the toxicity effects against tumor cells in-vitro, however, the immunotoxin showed unspecific killing to non-target cells as due to the ability of equinatoxin II to bind generally to cell membranes. Though previous studies considered actinoporin as a potential toxin moiety for immunotoxin, the non-specific binding capability remains as the main issue that needs to be addressed immediately. In a more recent study, sticholysin I (StI) was conjugated to IOR-C5 targeted mAb to target colon tumor cell line [145]. In this case, the immunotoxin binds preferentially to the target cells through the mAb rather than the native toxin binding region.

The last challenge of therapeutic application of immunotoxins is the high immunogenicity of the toxin moiety over long-term or repeated applications. Various strategies including combination with immunosuppressive agents, polyethylene glycol modification and generation of mutant toxins to reduce the immunogenicity have been attempted [146]. Unfortunately, these strategies were shown to be less effective. Current strategy to reduce the immunogenicity of toxin moiety of immunotoxin can be done by B-cell or T-cell epitope mapping and mutations [147]. A number of bacterial PFTs-based immunotoxins have been proven to maintain low immunogenicity after mutations were introduced to the B-cell or T-cell epitopes [147,148,149]. For example, a mesothelin-targeting recombinant immunotoxin (LMB-T14), after incorporating multiple mutations into the toxin moiety PE38 (exotoxin of Pseudomonas), had both B- and T-cell epitopes suppressed but remained high cytotoxic to mesothelioma cells derived from patients and other cancer cell lines [147]. So far, de-immunization of immunotoxins has mainly been reported for bacterial toxin-based immunotoxins, but the same approach can be applied to deimmunize other therapeutic toxins. We shall also take note that the de-immunization reduce but does not totally remove the immunogenicity. Moreover, introducing mutations in B- or T-cell epitopes may create new epitopes and to the worst extent, it may alter the structure of protein. To overcome this, it is important to fully understand the molecular mechanism and signaling cascades underlying the cellular responses against toxins.

## Figures and Tables

**Table 1:** Summary of PFTs described in this article.

Pore Forming Toxin	Species Name	MW (kDa) ^a^	Type of PFT	Reference
α-Hemolysin (Hla)	*Staphylococcus aureus*	33.2	β	[15,16]
γ-Hemolysin (LukF and γHLII)	*Staphylococcus aureus*	34.3 and 32.5	β	[16]
Leukocidin (LukF and LukS)	*Staphylococcus aureus*	34.3 and 32.5	β	[16,17]
Colicin	*Escherichia coli*	60	α	[18]
Aerolysin	*Aeromonashydrophila*	52	β	[19,20]
α-Toxin	*Clostridiumsepticum*	46.5	β	[21]
Parasporin-2	*Bacillus thuringiensis*	37	β	[22]
Cry5B	*Bacillus thuringiensis*	140	α	[23]
Tetanolysin O	*Clostridium tetani*	55	β	[24]
Pneumolysin	*Streptococcus pneumonia*	52	β	[25]
Cytolysin (VCC)	*Vibrio cholera*	80	β	[26]
Exotoxin A	*Pseudomonas aeruginosa*	38	α	[27]
Diphtheria toxin	*Corynebacterium diphtheriae*	58.3	α	[28]
Cytolysin LSL	*Laetiporus sulphureus*	35	β	[29]
Sticholysin	*Stichodactyla heliantus*	20	α	[30]
Equinatoxin	*Actinia equina*	20	α	[31,32]
Fragaceatoxin C	*Actinia fragacea*	20	α	[33]
HALTs	*Hydra magnipapillata*	20	α	[34]
Hydralysin (Hln)	*Chlorohydra viridissima*	27	β	[35]
Nvlysin-1b	*Nematostella vectensis*	NA	β	[36]
CrTX-A	*Carybdea rastoni*	43	α	[37]
CrTX-B	*Carybdea rastoni*	46	α	[37]
CaTX-A	*Carybdea alata*	43	α	[38]
CaTX-B	*Carybdea alata*	45	α	[38]
CqTX-A	*Chiropsalmus quadrigatus*	44	α	[39]
MkTX-A	*Malo kingi*	48.55	α	[40]
MkTX-B	*Malo kingi*	43–46	α	[40]
PsTX-60A	*Phyllodiscus semoni*	60	β	[41]
PsTX-60B	*Phyllodiscus semoni*	60	β	[42]
AvTX-60A	*Actineria villosa*	60	β	[43]
HyMac	*Hydra magnipapillata*	NA	β	[44]
Apextrin	*Hydra magnipapillata*	NA	β	[44]
CfTX-1	*Chironex fleckeri*	51.4	α	[45]
CfTX-2	*Chironex fleckeri*	51.7	α	[45]
CfTX-A	*Chironex fleckeri*	40	α	[46]
CfTX-B	*Chironex fleckeri*	42	α	[46]
RTX-A	*Heteractis crispa*	20	α	[47]

^a^ NA indicates the molecular weight of PFT is not available in current literature.
